# Deciphering the Role of Rapidly Evolving Conserved Elements in Primate Brain Development and Exploring Their Potential Involvement in Alzheimer's Disease

**DOI:** 10.1093/molbev/msae001

**Published:** 2024-01-04

**Authors:** Benxia Hu, Xiao-Lin Zhuang, Long Zhou, Guojie Zhang, David N Cooper, Dong-Dong Wu

**Affiliations:** Key Laboratory of Genetic Evolution & Animal Models, Kunming Natural History Museum of Zoology, Kunming Institute of Zoology, Chinese Academy of Sciences, Kunming, Yunnan, China; Key Laboratory of Genetic Evolution & Animal Models, Kunming Natural History Museum of Zoology, Kunming Institute of Zoology, Chinese Academy of Sciences, Kunming, Yunnan, China; Center of Evolutionary and Organismal Biology, and Women’s Hospital, Zhejiang University School of Medicine, Hangzhou, Guangdong, China; Liangzhu Laboratory, Zhejiang University Medical Center, Hangzhou, Guangdong, China; Center of Evolutionary and Organismal Biology, and Women’s Hospital, Zhejiang University School of Medicine, Hangzhou, Guangdong, China; Liangzhu Laboratory, Zhejiang University Medical Center, Hangzhou, Guangdong, China; Institute of Medical Genetics, School of Medicine, Cardiff University, Cardiff, UK; Key Laboratory of Genetic Evolution & Animal Models, Kunming Natural History Museum of Zoology, Kunming Institute of Zoology, Chinese Academy of Sciences, Kunming, Yunnan, China; National Resource Center for Non-Human Primates, Kunming Primate Research Center, and National Research Facility for Phenotypic and Genetic Analysis of Model Animals (Primate Facility), Kunming Institute of Zoology, Chinese Academy of Sciences, Kunming, Yunnan, China

**Keywords:** RECE, *cis*-regulatory element, enhancer, neurodevelopment

## Abstract

Although previous studies have identified human-specific accelerated regions as playing a key role in the recent evolution of the human brain, the characteristics and cellular functions of *rapidly evolving conserved elements* (RECEs) in ancestral primate lineages remain largely unexplored. Here, based on large-scale primate genome assemblies, we identify 888 RECEs that have been highly conserved in primates that exhibit significantly accelerated substitution rates in the ancestor of the Simiiformes. This primate lineage exhibits remarkable morphological innovations, including an expanded brain mass. Integrative multiomic analyses reveal that RECEs harbor sequences with potential *cis*-regulatory functions that are activated in the adult human brain. Importantly, genes linked to RECEs exhibit pronounced expression trajectories in the adult brain relative to the fetal stage. Furthermore, we observed an increase in the chromatin accessibility of RECEs in oligodendrocytes from individuals with Alzheimer's disease (AD) compared to that of a control group, indicating that these RECEs may contribute to brain aging and AD. Our findings serve to expand our knowledge of the genetic underpinnings of brain function during primate evolution.

## Introduction

The highly integrated brain structures and excellent cognitive abilities are the most distinctive features of primates, particularly humans. Elucidating the genetic mechanisms that have driven primate brain evolution has long been a central issue in evolutionary biology. Nearly 50 years ago, King and Wilson (1975) hypothesized that phenotypic differentiation among primate relatives could result from differences in *cis-regulatory elements* (CREs), including promoters and enhancers. Many subsequent studies have provided evidence for the contention that differences in CREs between human and nonhuman primates have been functionally significant in the evolution of the primate brain (Boyd et al. 2015; Vermunt et al. 2016; Jeong et al. 2021; Liu et al. 2021). For example, Pollard et al. identified human accelerated regions (HARs) in the human genome that, despite being evolutionarily conserved across the vertebrates, have diverged specifically in humans and found that they may function in brain development (Pollard et al. 2006; Hubisz and Pollard 2014). Recent studies have shown that nearly half of all HARs in the human genome harbor enhancer activity in the brain (Girskis et al. 2021; Keough et al. 2023). In addition, studies have demonstrated that mutations in HARs may be linked to a number of human brain disorders, including schizophrenia (SCZ) and autism spectrum disorder (ASD) (Oksenberg et al. 2013; Xu et al. 2015; Doan et al. 2016). Most of these studies have focused on the human lineage; however, our recent work has shown that, with the exception of the lineages of *Homo sapiens* and Hominidae, the ancestor of Simiiformes was also a key lineage with respect to the rapid expansion of the brain during primate evolution (Shao et al. 2023; Zhuang et al. 2023). In addition, it should be appreciated that the evolution of the primate brain has been a continuous process, the marked expansion of the neocortex (NCX) and the higher cognitive ability of humans gradually evolving from our remote primate ancestors. Therefore, we reasoned that it is necessary to investigate genetic innovations, particularly those involving rapidly evolving regulatory elements in the primitive primate lineages, which promise to provide new insights into primate brain evolution.

In order to comprehensively identify rapidly evolving regulatory elements from primates and to ascertain their functions in both brain development and inherited disease, we have applied a comparative genomics approach to search for *rapidly evolving conserved elements* (RECEs) across the primates, yielding 888 RECEs that were highly conserved in other primates but which showed evidence of significantly accelerated substitution rates in the ancestor of the Simiiformes. Next, we utilized available DNase-Seq (DNase I hypersensitive sites sequencing) data from the Epigenomics Roadmap (Kundaje et al. 2015) to assay regulatory functions of RECEs in primary cell types and tissues. Consistently, approximately 20% of our RECEs were also found to be highly enriched in DNase I hypersensitive sites (DHSs) in human fetal and adult brain (Girskis et al. 2021), indicating that these RECEs may act as enhancers or promoters to activate gene expression. By integrating chromatin loops with H3K27ac chromatin immunoprecipitation followed by sequencing (ChIP–seq) data, we were able to predict the putative target genes of RECEs in human fetal and adult brain tissue, respectively. For example, the target genes of RECEs detected in neurons and glia from the human adult brain showed cell-type–specific expression based on single-nucleus RNA-seq (snRNA-seq) data (Lake et al. 2016). Based on the coexpression network analysis of human brain developmental transcriptomes (Zhu et al. 2018), we observed that certain genes targeted by RECEs in human fetal and adult brains were significantly related to the human adult brain development. Moreover, the genes targeted by RECEs exhibited an unconventional developmental pattern in which their expression levels gradually increased from embryo to adulthood. We further observed that chromatin accessibility was increased in oligodendrocytes in individuals with Alzheimer's disease (AD) compared to the control group, suggesting that RECEs could be a contributory cause of AD.

## Results

### Characterization of RECEs in the Ancestor of the Simiiformes

**Fig. 1.**
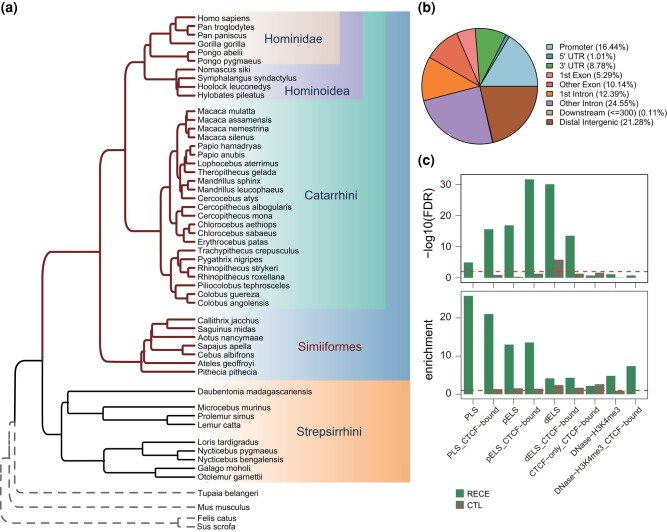
Characteristics of RECEs. a) Phylogenetic tree of primate genomes. b) Genomic distribution of RECEs. c) Enrichment analysis for the RECEs in the candidate CREs derived from ENCODE data (Moore et al. 2020). PLS, promoter-like; PLS_CTCF-bound, promoter-like&CTCF; pELS, proximal enhancer-like; pELS_CTCF-bound, proximal enhancer-like&CTCF; dELS, distal enhancer-like; dELS_CTCF-bound, distal enhancer-like&CTCF; CTCF-only_CTCF-bound, CTCF-only; DNase-H3K4me3, DNase-H3K4me3; DNase-H3K4me3_CTCF-bound, DNase-H3K4me3&CTCF.

RECEs are conserved elements that have experienced rapid evolution in specific lineages. Our previous studies have identified many RECEs across the primates and revealed their potential functions in evolution (Bi et al. 2023; Zhuang et al. 2023). Here, to investigate the characteristics and functions of RECEs in the ancestor of Simiiformes, we applied comparative genomics to define their mutational landscape across different primates, identifying 888 RECEs in the ancestor of the Simiiformes (Fig. 1a; supplementary table S1a and b and fig. S1a and b, Supplementary Material online; Materials and Methods). Given that the relative brain volume increased significantly in the ancestor of Simiiformes by comparison with the more “primitive” primates, such as lemurs and lorises (Shao et al. 2023), and previously published research evidenced that the rapidly evolved conserved noncoding elements in the ancestor of Simiiformes could drive the rapid expansion of the brains (Zhuang et al. 2023), we sought to further explore the evolutionary changes that impacted gene regulatory elements from the most primitive primate lineage of the Strepsirrhini to the Simiiformes (Fig. 1a).

Here, we first compared reported HARs (Girskis et al. 2021) with our RECEs. The median size of the RECEs was 190 bp (range: 24 to 2,448 bp), which was considerably smaller than that of the HARs (269 bp). The median GC content of these RECEs was 0.14 (range: 0.14 to 0.81), which was comparable to that of the HARs (supplementary fig. S1c and d, Supplementary Material online). As expected, only 2 RECEs overlapped with HARs. We further checked the genomic distributions of the RECEs and found that they were mostly located within intronic regions, followed by distal intergenic regions and promoters (Fig. 1b) (Girskis et al. 2021), indicating that RECEs could be involved in the transcriptional regulation of target genes.

To further explore the functions of RECEs, we sought to define the epigenetic properties of these RECEs based on candidate CREs derived from ENCODE data (Moore et al. 2020) and observed that RECEs were significantly enriched in gene promoters and distal enhancers compared with size-matched random sequences (CTL) (Fig. 1c). Moreover, RECEs also exhibited a high degree of enrichment for CTCF-binding sites (e.g. dELS_CTCF-bound) (Fig. 1c). Keough et al. (2023) have reported that human-specific substitutions in HARs alter transcription factor-binding sites. Next, we wanted to examine whether RECEs could contribute to transcription factor-binding sites. To address this question, we ran ReMapEnrich (Chèneby et al. 2020) and AnimalTFDB Database (http://bioinfo.life.hust.edu.cn/AnimalTFDB#!/) (Hu et al. 2019) to evaluate the enrichment of RECEs for transcription factor-binding sites and found that several transcription factors, such as CTCF, SMC3, RAD21, and SPI1 (PU.1), were significantly enriched within RECEs (supplementary table S2a and b, Supplementary Material online). For example, CTCF and both SMC3 and RAD21 that are central components of Cohesin display critical roles in gene transcription and 3D genome architecture (Losada et al. 1998; Dixon et al. 2012; Nora et al. 2012; Rao et al. 2014), suggesting that RECEs, alongside CTCF- and RAD21-binding sites, possess the capability to shape 3D genome organization. The deletion of *CTCF* in the mouse brain gives rise to defective dendritic arborization and spine density during brain development (Hirayama et al. 2012). The deletion of *SMC3* in the mouse brain causes defective synapse development and anxiety-related behavior (Fujita et al. 2017), whereas SPI1 (PU.1) is known to regulate the expression of AD risk genes in primary human microglia (Smith et al. 2013; Rustenhoven et al. 2018). These results further support the postulate that transcription factors bind to RECEs.

### RECEs Are Enriched for CREs in the Human Brain

**Fig. 2.**
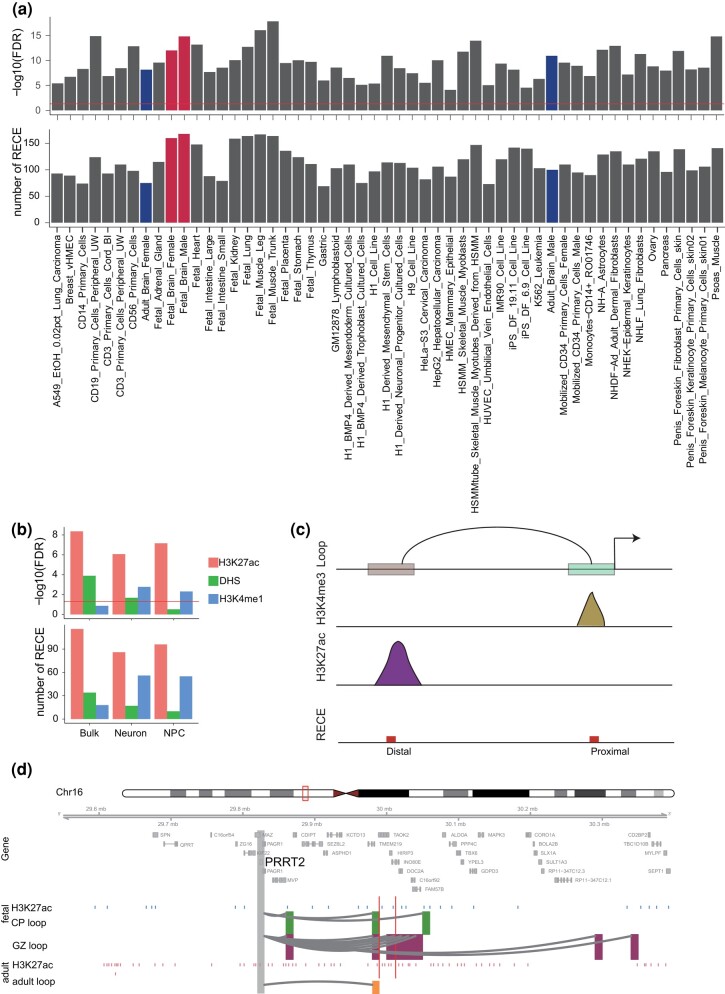
Analysis of chromatin accessibility of RECEs in the human brain. a) Chromatin accessibility of RECEs in human primary cells and tissues. The fetal and adult brains are highlighted in red and blue, respectively. b) Chromatin accessibility of RECEs in bulk fetal human brain tissues, neuron, and NPC, respectively. c) Diagram depicts how to link RECEs to proximal and distal CREs. d) A neuronal gene, *PPRT2*, is engaged with fetal- (CP and GZ) and adult-specific H3K27ac peaks via enhancer–promoter interactions. The regions that interact with the gene promoter (gray) are highlighted in green (CP), purple (GZ), and yellow (adult). Blue and pink bars represent fetal and adult-specific H3K27ac peaks. Red bars represent RECEs.

To examine whether our RECEs harbor features of in vivo regulatory activity in the human brain, we tested the chromatin state at RECEs across diverse primary cell types and tissues, including fetal and adult, using DNaseI hypersensitivity mapping (DNaseI-seq data) (Kundaje et al. 2015). Our results revealed that 17% of RECEs exhibited chromatin accessibility in human brain tissue (Fig. 2a). Consistent with previous studies (Girskis et al. 2021), many more RECEs were preferentially accessible in human fetal brain tissue than in adult human brain tissue (Fig. 2a), indicating that our RECEs may serve as neurodevelopmental regulatory elements with a role in human brain development. The next question was whether RECEs act as cell-type–specific enhancers in the maturing neurons and neural progenitor cells (NPCs) isolated from the human fetal brain. To address this question, we determined the overlap between RECEs and H3K4me1 and/or H3K27ac ChIP-seq peaks evident in NPCs or maturing neurons (Girskis et al. 2021) and noted 150 RECEs that displayed enhancer-like features in NPCs and maturing neurons consistent with their possessing in vivo enhancer activity (Fig. 2b). Therefore, our results indicate that RECEs may act as neurodevelopmental enhancers in the human fetal brain.

To predict the putative target genes of RECEs in the human fetal and adult brain, respectively, we integrated Hi-C with H3K4me3 and H3K27ac ChIP-seq data to map the RECEs to active gene promoters and promoter-interacting distal enhancers. We noted that 30 RECEs interacted with 76 cognate genes in the human fetal brain and that 85 RECEs, overlapping with active gene promoters defined by human fetal brain H3K4me3 ChIP-seq data, might regulate 90 genes (Fig. 2c). Both CP and GZ RECE-associated genes were significantly enriched for gene ontology (GO) terms related to axon, synapse, and protein kinase complex (supplementary fig. S2a and b and table S3, Supplementary Material online). In the human adult brain, 87 RECEs looped to promoters of 137 cognate genes, whereas 61 RECEs overlapping with active gene promoters might activate 66 genes (Fig. 2c). Adult RECE-associated genes were significantly involved in the cell cycle and the protein kinase complex (supplementary fig. S2c and table S3, Supplementary Material online). For example, *PRRT2* is associated with a range of paroxysmal neurological disorders (Wang et al. 2011; Landolfi et al. 2021) and affects synaptic functions (Valtorta et al. 2016; Coleman et al. 2018). *PRRT2* was highly expressed in human brain tissue, especially in the cerebellum (supplementary fig. S2d, Supplementary Material online). Chromatin looping confirmed that 1 RECE (chr16: 30012469 to 30012931) overlapped with the *PRRT2* promoter-interacting enhancer region (Fig. 2d), suggesting that this RECE may control *PRRT2* expression in the human fetal and adult brain.

### RECE Target Genes Display Increasing Expression Trajectories in Adult Brain

**Fig. 3.**
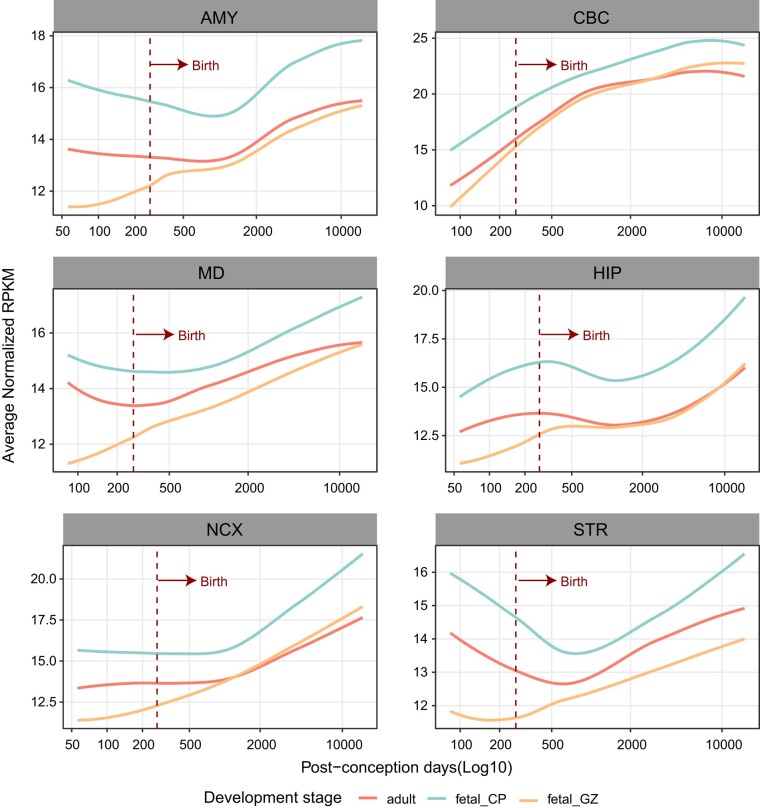
The expression trajectories of RECE-associated genes in the fetal and adult human brain. Expression trajectories of genes targeted by RECEs in human fetal and adult brains during the course of human brain development. The expression level of genes in samples at specific developmental time points is represented by the median expression level with correction against the background of all expressed genes in human brain transcriptomes. The expression data were obtained from previous studies (Zhu et al. 2018).

To explore whether RECEs are involved in the gene regulatory networks during brain development, we used the genes linked to RECEs (hereafter referred to as RECE genes) in the human fetal and adult brain to separately construct gene coexpression networks in 6 human brain regions (amygdala [AMY], striatum [STR], hippocampus [HIP], mediodorsal nucleus of the thalamus [MD], NCX, and cerebellar cortex [CBC]) ranging from the embryonic period to adulthood (Zhu et al. 2018). The results showed that some coexpression modules, constructed by RECE genes from fetal and adult brains, were significantly related to human brain development (supplementary table S4, Supplementary Material online; a module was determined to significantly relate to brain development if the absolute value of the module-trait correlation coefficient was >0.5 and significance was <0.05). In contrast to the traditional developmental patterns of certain genes that show their highest levels of expression during the embryonic period (Domazet-Loso et al. 2007), the developmental trajectories of RECE genes in 6 brain regions indicated that there may be a positive correlation between RECE genes and human brain development (Fig. 3). In summary, our findings indicate that RECEs play a role in the maintenance of adult brain functions.

### RECEs Target Genes Display Cell-Type–Specific Functions in the Human Adult Brain

**Fig. 4.**
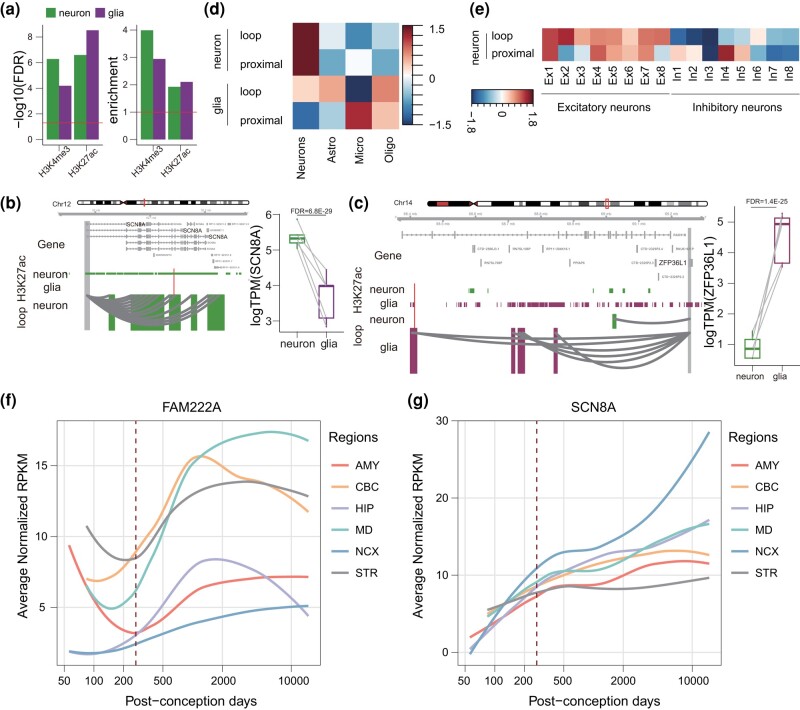
RECE target genes display cell-type–specific functions in the human adult brain. a) Analysis of regulatory activity of RECEs in the neurons and glia from adult human brains based on H3K4me3 and H3K27ac ChIP-seq data. b, c) A neuronal gene, *SCN8A*, is engaged with neuron-specific H3K27ac peaks via enhancer–promoter interactions in neurons b), while a glial-expressed gene, *ZFP36L1*, is engaged with glia-specific H3K27ac peaks via enhancer–promoter interactions in glia c). The regions that interact with the gene promoter (gray) are highlighted in green (neuron), and purple (glia). Red bars represent RECEs. Boxplots on the right show expression levels of *SCN8A* and *ZFP36L1* in neurons and glia. The FDR was calculated by DESeq2. d) Genes assigned to neuron-specific peaks are highly expressed in neurons, whereas genes assigned to glia-specific peaks are highly expressed in microglia and oligodendrocytes. e) Neuronal subtype expression profiles of RECE-associated genes. f, g) Expression trajectories of *FAM222A* and *SCN8A* in human fetal and adult brains during the course of human brain development. The expression level of a sample at a specific developmental time point is represented by the median expression level with correction against the background of all expressed genes in human brain transcriptomes. The expression data were obtained from previous studies (Zhu et al. 2018). Astro, astrocytes; Endo, endothelial; Micro, microglia; Oligo, oligodendrocytes.

There were 100 and 75 RECEs overlapping with DNaseI hypersensitive sites in the human adult female and male brains, respectively (Fig. 2a), indicating that these RECEs should also function as regulatory elements in the human adult brain. We have built the cell-type–specific gene regulatory maps for neurons and glia in the human adult brain (Hu et al. 2021). Therefore, we sought to identify their functions in 2 major cell types, neurons and glia, in the human adult brain. We observed that RECEs were highly enriched in cell-type–specific active promoters and enhancers defined by H3K4me3 and H3K27ac ChIP-seq data in neurons and glia (Girdhar et al. 2018) (Fig. 4a). Here, we used neuronal and glial H3K4me3 ChIP-seq data that served to define active promoters of genes overlapping with RECEs and obtained neuronal and glial RECE-proximal genes, respectively (supplementary table S5, Supplementary Material online). Neuronal RECE-proximal genes were significantly enriched for GO terms related to the protein kinase complex (False Discovery Rate [FDR] = 0.003) (Bennison et al. 2020), including *CDK12* (Chen et al. 2017), whereas glial RECE-proximal genes were involved in nucleolar organization (FDR = 0.04), indicating that glial RECE-proximal genes may play a role in regulating neuronal gene expression. In order to predict putative RECE-regulated genes through long-range enhancer–promoter interactions in the human adult brain, we identified RECEs overlapping with neuronal and glial promoter-anchored enhancers, respectively. We were able to assign 88 and 72 RECEs to 242 and 194 genes (referred to as neuronal and glial RECE-loop genes, respectively) via chromatin interactions in neurons and glia (supplementary table S5, Supplementary Material online). Linking RECEs to cell-type–specific enhancer–promoter interactions revealed specific features of RECEs that pertain to the regulation of cell-type–specific gene expression. For example, *SCN8A*, the mutation of which is a cause of severe intellectual disability and autism (Liu et al. 2019), was engaged in RECE and neuron-specific loops whereas its expression level was significantly higher in neurons compared to glia (Fig. 4b). *ARF1*, a critical regulator of synaptic function (Rocca et al. 2013), was engaged in a RECE and neuron-specific loops, whereas its expression level was significantly higher in neurons compared to glia (supplementary fig. S3a and b, Supplementary Material online). *ZFP36L1* is necessary for the oligodendrocyte–astrocyte lineage transition (Weng et al. 2019). We noted that *ZFP36L1* was engaged in a RECE and glial-specific loops, while its expression was significantly higher in glia as compared with neurons (Fig. 4c). Moreover, consistent with our previous results (Hu et al. 2021) based on single-cell RNA-seq data (Darmanis et al. 2015), neuronal RECE-loop genes and RECE-proximal genes were more highly expressed in neurons. By contrast, glial RECE-proximal genes were highly expressed in microglia, and glial RECE-loop genes were highly expressed in oligodendrocytes and astrocytes (Fig. 4d), demonstrating the tight relationship between cell-type–specific RECEs and the expression signature. Neuronal subtype expression profiles of RECE-loop and RECE-proximal genes were evaluated using snRNA-seq data obtained from excitatory (Ex1 to Ex8) and inhibitory (In1 to In8) neuronal subtypes (Lake et al. 2016). RECE-loop genes displayed relatively higher expression signatures in L3/4 neurons (Ex2) and L2/3 cortical projection neurons (Ex1). RECE-proximal genes displayed relatively higher expression signatures in subcortical projection neurons (Ex4) and L2/3 cortical projection neurons (Ex1). In interneurons, RECE-proximal genes showed significant enrichment in VIP − RELN + NDNF + (In4) (Fig. 4e). In summary, our results confirmed that our RECEs display cell-type–specific CREs in the human adult brain.

Based on human brain development transcriptomes (Zhu et al. 2018), we analyzed the developmental trajectories of neuronal RECE-proximal genes, glial RECE-proximal genes, neuronal RECE-loop genes, and glial RECE-loop genes in 6 brain regions. Interestingly, the expression levels of neuronal and glial RECE-proximal genes continued to increase from the embryonic period into adulthood (supplementary fig. S5, Supplementary Material online), suggesting that RECE-proximal genes might be critical for human brain development. In contrast to RECE-proximal genes, the expression levels of neuronal and glial RECE-loop genes exhibited few changes during development. However, some RECE-loop genes displayed dysregulation during brain development between embryo and adulthood (Fig. 4f and e). *FAM222A*, a gene putatively involved in brain atrophy susceptibility but also closely associated with AD (Satoh et al. 2020; Yan et al. 2020), was one of the glial RECE-loop genes; its expression during brain development was reduced between embryo and birth but was significantly induced after birth (Fig. 4f). Another example, *SCN8A*, one of the neuronal RECE-loop genes, displayed an increase in expression throughout development (Fig. 4e). Selective targeting of *Scn8a* prevented seizures in the mouse model of medial temporal lobe epilepsy (Wong et al. 2018). The above results suggest that RECEs played an important role in adult brain development and probably also in the etiology of neurological disease.

### RECEs Are Potentially Involved in the Etiology of AD

**Fig. 5.**
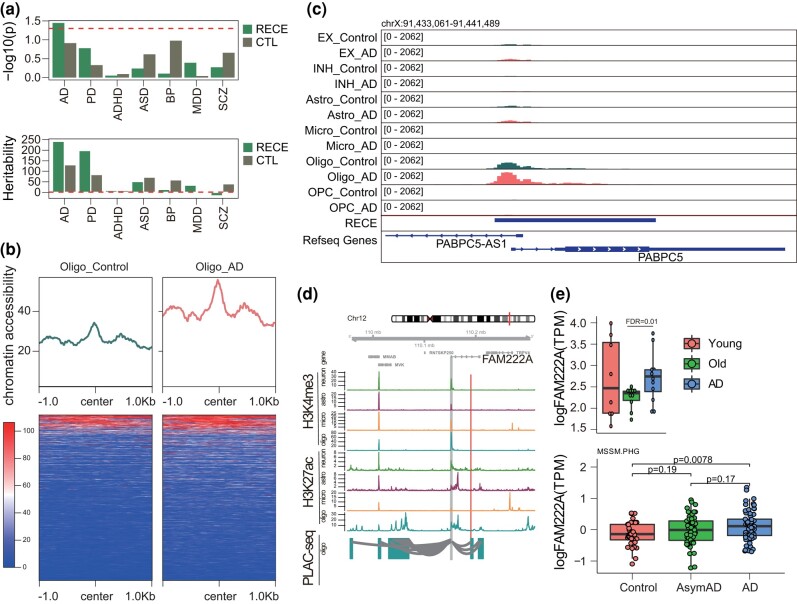
RECEs are potentially involved in the etiology of AD. a) Heritability enrichment of human brain disorders GWAS in RECEs. b) Chromatin accessibility of RECEs in oligodendrocytes based on snATAC-seq from individuals with and without AD. c) The genomic track showing a RECE overlapping with the oligodendrocyte ATAC-seq peak. d) An AD risk gene, *FAM222A*, is engaged with oligodendrocyte-specific H3K27ac peaks via enhancer–promoter interactions. The regions that interact with the gene promoter (gray) are highlighted in blue (oligo). Red bars represent RECEs. e) (Top panel) Expression levels of *FAM222A* in young, old, and AD human brains (FDR is calculated by DESeq2), and (Bottom panel) Expression levels of FAM222A in control, AsymAD, and AD brain tissues (http://swaruplab.bio.uci.edu:3838/bulkRNA/).

Genome-wide association studies (GWAS) have identified many genetic variants associated with a highly diverse range of diseases. Previous studies have reported that more than 90% of genetic variants are enriched in noncoding regions (Frydas et al. 2022). Given that CNEs can act as CREs such as enhancers, we posed the question as to whether our CNE list was enriched in any human brain disease-associated genetic variants, especially single-nucleotide polymorphisms (SNPs). To link RECEs to causal genetic factors, we next determined the enrichment of AD, Parkinson's disease (PD), ASD, attention deficit/hyperactivity disorder (ADHD), bipolar disorder (BD), major depression disorder (MDD), and SCZ-associated common genetic variants in RECEs. AD SNP-based heritability was highly enriched in RECEs (*P* = 0.036), suggesting that RECEs may contribute to the genetic etiology of AD (Fig. 5a). Unexpectedly, we observed that the increased chromatin accessibility was only observed in oligodendrocytes from individuals with AD compared to that of a control group based on single-nucleotide assays for transposase-accessible chromatin sequencing (snATAC-seq) data (Fig. 5b and c; supplementary fig. S5a, Supplementary Material online), which indicates that these RECEs may contribute to brain aging and the etiology underlying AD. Next, we wanted to test the distribution of RECEs in active *CRE*s (promoter and enhancer) in glia (astrocytes, microglia, and oligodendrocytes) (Nott et al. 2019). We observed that RECEs were highly enriched in glial promoters and enhancers (supplementary fig. S5b, Supplementary Material online). To test whether distal RECEs interacted with the promoters of AD risk genes, we integrated proximity ligation-assisted ChIP-seq (PLAC-seq) with H3K27ac ChIP-seq data in the human brain (supplementary fig. S5c and table S6, Supplementary Material online). One RECE (chr12: 110190581 to 110190690) overlapped with an oligodendrocyte H3K27ac peak interacting with the *FAM222A* promoter, indicating that this RECE may control the expression of *FAM222A* a well-known AD risk gene (Satoh et al. 2020; Yan et al. 2020) (Fig. 5d), which was upregulated in the AD patients compared to controls (Fig. 5e). We further observed that *FAM222A* was cell type specifically expressed in oligodendrocytes based on snRNA-seq (Morabito et al. 2021) (supplementary fig. S5d, Supplementary Material online). Another example, the RECE (chr11: 6566819 to 6568345) only overlapped with the oligodendrocyte H3K27ac peak that looped to *TPP1* based on PLAC-seq data (supplementary fig. S6a, Supplementary Material online). The snATAC-seq data further showed that this RECE only intersected with an open chromatin region detected in oligodendrocytes (supplementary fig. S6b, Supplementary Material online). TPP1 can degrade fibrillar forms of amyloid-beta via endopeptidase (Solé-Domènech et al. 2018). Therefore, our results suggest that this RECE may control *TPP1* expression. Owing to the sequence nature of snRNA-seq, *TPP1* was not detected by snRNA-seq (Morabito et al. 2021). However, based on bulk RNA-seq from the Mayo.TC database, *TPP1* was increased in AD compared to controls (supplementary fig. S6c, Supplementary Material online). Taken together, we surmise that RECEs may be involved in AD pathogenesis by regulating the expression of AD risk genes.

## Discussion

Over the years, many studies have demonstrated that changes of gene regulatory elements in the human lineage have driven the rapid evolution of the human brain (Jeong et al. 2021; Prabhakar et al. 2006; Pollard et al. 2006). It has been further shown that these rapidly evolving regulatory elements function mainly in the embryonic brain during early human development (Pollard et al. 2006; Luo et al. 2021; Reilly et al. 2015; Florio et al. 2015; Silbereis et al. 2016; Boyd et al. 2015; Hou et al. 2021). Recently, our studies have indicated that RECEs have also played an important role in the evolution of other primates, especially in the ancestor of the Simiiformes (Shao et al. 2023; Zhuang et al. 2023). In this study, we comprehensively investigated the neurological functions of RECEs in the Simiiformes in terms of the evolutionary development of the primate brain. Our results show that RECEs may be involved in the development of the adult brain, consistent with the view that RECEs are crucial for the establishment of the advanced cognitive abilities characteristic of the primates and potentially important for our emerging understanding of the pathogenesis of human neurological disease.

Previous studies have identified human-specific accelerated regions as being involved in brain development (Pollard et al. 2006; Hubisz and Pollard 2014; Keough et al. 2023; Girskis et al. 2021). However, the comprehensive identification of RECEs in other primate lineages and their functional roles in both brain development and disease pathogenesis have been unclear. By interrogating the epigenetic landscape of 888 RECEs in the ancestor of the Simiiformes across different primary tissues and cells, we observed that approximately 20% of RECEs exhibit high chromatin accessibility. By integrating our RECEs with chromatin data from developing fetal and adult brains, we established that a substantial portion of RECEs may act as in vitro and in vivo promoters or enhancers (Pollard et al. 2006; Hubisz and Pollard 2014; Girskis et al. 2021; Wittkopp and Kalay 2011). Hence, we hypothesize that RECEs represent genomic entities that have driven Simiiformes-specific neurodevelopmental gene regulatory programs. Consistent with this postulate, the expression levels of genes associated with RECEs were noted to gradually increase from the embryonic stage to adult, which may be indicative of their playing a role in promoting adult brain development. Genetic mutations within human-specific accelerated regions may also contribute to human brain developmental disorders (Oksenberg et al. 2013; Xu et al. 2015; Doan et al. 2016). Based on Linkage disequilibrium score regression analysis, our RECEs are only enriched for AD-associated common genetic variants (SNPs) (Bulik-Sullivan et al. 2015). Many previous studies have reported that AD-associated SNPs are enriched in glial, especially microglial enhancer regions (Nott et al. 2019; Hu et al. 2021; Morabito et al. 2021; Li et al. 2023). As shown in supplementary fig. S5a, Supplementary Material online, the chromatin accessibility of RECEs was increased in microglia from human AD brains as compared to those from human normal brains. Moreover, the chromatin accessibility of RECEs was not changed between excitatory and inhibitory neurons from human AD brains compared to those from human normal brains. That is one possible reason why our RECEs are only enriched in AD-associated SNPs. Another possible reason is that the number of RECEs is low. In addition, we discovered that RECEs exhibit significantly higher chromatin accessibility in oligodendrocytes among individuals with AD than in individuals without AD. Therefore, RECEs not only appear to play important roles in adult brain development but also potentially in the pathogenesis of AD. However, the number of open chromatin regions in oligodendrocytes overlapping with RECEs was too small, which may be due to the sparsity of snATAC-seq data (Cusanovich et al. 2015). We obtained roughly 20% overlap between RECEs and the active promoter and enhancer regions in oligodendrocytes as defined by H3K4me3 and H3K27ac ChIP-seq data, respectively (Fujita et al. 2017). One way to validate this finding would be to perform bulk ATAC-seq in oligodendrocytes from individuals with and without AD. Previous studies have found that human-specific accelerated regions are enriched in the domain of human-specific structural variants and serve to alter 3D genome folding, including topologically associating domain (Dixon et al. 2012; Nora et al. 2012) and enhancer–promoter interactions (Bonev and Cavalli 2016), in the process rewiring evolutionarily conserved enhancers to distinct target genes and regulatory domains (Keough et al. 2023). Given that our RECEs are enriched in CREs and contain CTCF- and RAD21-binding sites (Losada et al. 1998; Dixon et al. 2012; Nora et al. 2012; Rao et al. 2014), we propose that RECEs are capable of shaping 3D genome organization. In order to elucidate the molecular mechanisms by which RECEs exert their effect, genome-editing techniques like CRISPR-Cas9 could be employed. Specifically, deleting select RECEs in iPSC-derived oligodendrocytes could provide insights into whether or not these RECEs influence AD risk genes.

In summary, this study has extensively explored the potential roles of Simiiformes RECEs in adult brain development and neurodegenerative disease, thereby corroborating the view that the advanced cognitive abilities of the human brain have gradually evolved from the brains of our primate ancestors. Furthermore, it identifies certain RECEs as potential candidates for future investigations of human neurodegenerative disease.

## Materials and Methods

### Identification of RECEs

We employed 49 primate genome sequences from our previous research (Shao et al. 2023), with the exception of the Western tarsier (*Cephalopachus bancanus*). *C. bancanus* belongs to the lineage of Tarsiiformes that lies somewhere between the Strepsirrhini and the Simiiformes. Since the Tarsiiformes lineage did not show any marked changes in brain evolution according to previous research (Zhuang et al. 2023), we removed the Tarsiiformes lineage from our analysis for the sake of convenience. Based on 49-way primate genome alignments, we first filtered the alignments such that at least 90% of the species were present in the alignments. The highly conserved elements (HCEs) were predicted in primates using the phastCons program in PHAST (v1.5) (Hubisz et al. 2011) with options “--most-conserved --score options”. Next, the RECEs in the ancestor of Simiiformes were identified from HCEs. We then estimated acceleration scores for the HCEs using the phyloP program in PHAST (v1.5) (Hubisz et al. 2011) with the parameters “--method LRT, --branch and --mode ACC” for the ancestor of Simiiformes. The significant acceleration at FDR-adjusted *P*-values of ≤0.05 was considered in further analyses. RECEs in the Y chromosome were removed.

### Characterization of RECEs

To examine the characteristics of those RECEs we identified, we calculated the length and GC content of RECEs. We further ran ChIPseeker (v.1.26.2) (Yu et al. 2015) to check the genomic distributions of RECEs.

### LDSC Regression Analysis

We used LD score regression (LDSC) software (v.1.0.0) (Bulik-Sullivan et al. 2015) to assess the enrichment of the single-nucleotide variant-based heritability (*h*^2^) for brain disorder GWAS in the RECEs we identified. We first downloaded the following GWAS summary data sets: AD (*n* = 71,880 cases; 383,378 controls) (Jansen et al. 2019), PD (*n* = 37,688 cases; 1.4 million controls) (Nalls et al. 2019), ASD (*n* = 18,381 cases; 27,969 controls) (Grove et al. 2019), ADHD (*n* = 20,183 cases; 35,191 controls) (Demontis et al. 2019), BD (*n* = 41,917 cases; 371,549 controls) (Mullins et al. 2021), MDD (*n* = 246,363 cases; 561,190 controls) (Howard et al. 2019), and SCZ (*n* = 22,778 cases; 35,362 controls) (Lam et al. 2019). Genetic variants were annotated to RECEs, and SNP-based heritability statistics were calculated using the GWAS summary statistics mentioned above. Enrichment statistics were calculated as the proportion of SNP-based heritability divided by the proportion of SNPs annotated to RECEs.

### Enrichment of RECEs within Functional Genomic Annotations

To determine the enrichment of RECEs within functionally annotated genomic regions (Moore et al. 2020), we performed a binomial test (*pbinom()*) to delineate the epigenetic properties of RECEs and size-matched random sequences (CTL). *P*-values were adjusted by *FDR* (*p.adjust*). To test the enrichment of RECEs in DHSs, as well as enhancer and promoter regions in different tissues or cell types (Kundaje et al. 2015; Girdhar et al. 2018; Nott et al. 2019), we randomly selected 1,000 sets of DNA sequences (henceforth referred to as background regions, CTL) that were size matched with respect to the RECEs. We then performed Fisher's exact test (1,000 permutations) to calculate the enrichment (*P*-value and odd ratio) of RECEs overlapping with DHS enhancer and promoter regions in each tissue or cell type. Finally, we calculated the average *P*-values and odds ratios for the RECEs overlapping these DHS enhancer and promoter regions in different tissues or cell types.

### Identification of RECE Target Genes

Given that RECEs were mainly located within promoters and enhancers, we wanted to check the enrichment of RECEs in active promoters as predicted by H3K4me3 and promoter-interacting distal enhancers overlapping with chromatin loops. To address these 2 questions, we first downloaded H3K4me3 and H3K27ac ChIP-seq peaks and chromatin loops from Girdhar et al. (2018) and Nott et al. (2019), respectively. Gene promoters were defined as DNA sequence 2-kb upstream and 1-kb downstream of transcription start sites based on the human reference genome (Release26 [GRCh37]) from the GENCODE database. Active promoters were defined as gene promoters overlapping with H3K4me3 peaks. We used the intersection of active promoters and RECEs in order to identify RECE target genes (referred to as *RECE-proximal genes*). Promoter-interacting regions were defined based on their overlaps with gene promoter regions. The intersection of the RECEs with these promoter-interacting regions was used to define *RECE-loop genes*.

### RNA-seq Data Analysis

RNA-seq data for 8 young healthy brains (young), 10 aged healthy brains (old), and 12 aged diseased brains (AD) were downloaded from Nativio et al. (2018) (GSE104704). RNA-seq data for 5 paired Neuron and Glia from the BA9-dorsolateral prefrontal cortex were downloaded from Rizzardi et al. (2019) (GSE96613). We used FastQC (v.0.11.8) (https://www.bioinformatics.babraham.ac.uk/projects/fastqc/) to check the quality of the RNA-seq raw reads. The clean RNA-seq reads were mapped to the human reference genome (hg19) from the UCSC database with HISAT2 (v.2.2.1) (Satizabal et al. 2019) using default parameters. We assembled and quantified transcripts using StringTie (v.2.1.2) (Pertea et al. 2015). Differential analysis was done with DESeq2 (v.1.30.0) (Love et al. 2014). Normalized expression values (transcripts per million) of genes were compared across different conditions.

### snRNA-seq Data Analysis

We downloaded control and AD snRNA-seq data sets (Morabito et al. 2021) and used the cellranger count and aggr (v.7.0.0, 10× Genomics) with default parameters to align the snRNA-seq data to the GRCH37 human reference genome to produce the raw cell-by-gene count matrix based on the barcode matrix for all snRNA-seq libraries. Next, we used Seurat (v.4.3.0) (Hao et al. 2021) to filter out low-quality nuclei based on *nFeature_RNA > 200 & nFeature_RNA < 10000 & percent.mt < 10*. Subsequently, the merged expression matrix was normalized by the *SCTransform* with default parameters from Seurat. The principal components were calculated using the first 3,000 variable genes, and the Uniform Manifold Approximation and Projection (UMAP) analysis was performed with *RunUMAP* from Seurat. The *FindAllMarkers* from Seurat was used to analyze the differentially expressed genes across different clusters. Finally, we used the previously identified marker genes to define the different cell clusters or cell types based on Morabito et al. (2021).

### snATAC-seq Data Analysis

We downloaded control and AD snATAC-seq data sets (Morabito et al. 2021) and used the cellranger-atac count and aggr (v.2.1.0, 10× Genomics) with default parameters to align the snATAC-seq data to the GRCH37 human reference genome to produce the raw cell-by-peak matrix (single cell accessibility counts) based on the barcode matrix for all snATAC-seq libraries. Next, we performed an integrative analysis of the snRNA-seq and snATAC-seq by the *Signac* (v.1.10.0) from Seurat based on the approach of Morabito et al. (2021).

### WGCNA Analysis

Transcriptomes of developing human brains, ranging from the embryonic period to adulthood, were obtained from previous studies (Zhu et al. 2018). The various brain tissues examined included AMY, STR, HIP, MD, NCX, and CBC (Zhu et al. 2018). The coexpression network for samples of each brain tissue was constructed by WGCNA R packages (Langfelder and Horvath 2008), with normalized RPKM values serving as the input data for the WGCNA. To identify hub genes related to brain development in each module, we calculated module membership (MM) and gene significance (GS) for each gene in each module. MM is the correlation coefficient between expression values for each gene and “eigengene” (eigengene is the first principal component of each module) in each module. GS is the correlation coefficient between expression values and sample ages for each gene in each module. The gene with p.GS < 0.05 and p.MM < 0.05 was deemed to be the hub gene related to brain development in that module.

### GO Analysis

We applied gProfiler2 (Kolberg et al. 2020) to analyze the functional enrichment of genes of interest. Significant GO terms were selected based on an FDR < 0.05.

### Motif Analysis of Transcription Factor-Binding Sites

We ran ReMapEnrich (Chèneby et al. 2020) and AnimalTFDB Database (http://bioinfo.life.hust.edu.cn/AnimalTFDB#!/) (Hu et al. 2019) to predict transcription factor-binding sites in RECEs. The candidate transcription factor-binding sites predicted by ReMapEnrich were selected based on q.value < 0.01 and nb.overlaps > 1. We only kept the transcription factors predicted by the AnimalTFDB Database with the highest matched score and the adjusted *P*-value < 0.01.

## Supplementary Material

msae001_Supplementary_Data

## Data Availability

H3K4me3 and H3K27ac ChIP-seq peaks are available through (Girdhar et al. 2018; Nott et al. 2019). Young, old, and AD RNA-seq data are available through (Nativio et al. 2018). Neuronal, microglial, and oligodendrocyte PLAC-seq are available through (Nott et al. 2019). Human fetal and adult brain Hi-C data are available through (Won et al. 2016; Wang et al. 2018). Neuronal and glial Hi-C data are available through (Hu et al. 2021). Human fetal and adult snRNA-seq data are available through (Darmanis et al. 2015; Lake et al. 2016). AD snRNA-seq data are available through (Morabito et al. 2021). AD snATAC-seq data are available through (Morabito et al. 2021). HAR sequences are available through (Girskis et al. 2021).
