# Supplementary material for: Deciphering the Role of Rapidly Evolving Conserved Elements in Primate Brain Development and Exploring Their Potential Involvement in Alzheimer's Disease
Source: Mol Biol Evol. 2024 Jan 4;41(1):msae001. doi: 10.1093/molbev/msae001 (PMC10798191; doi:10.1093/molbev/msae001)
Supplement: msae001_Supplementary_Data [file msae001_supplementary_data.zip › Supplementary_Materials.docx]

**Figure S1**.


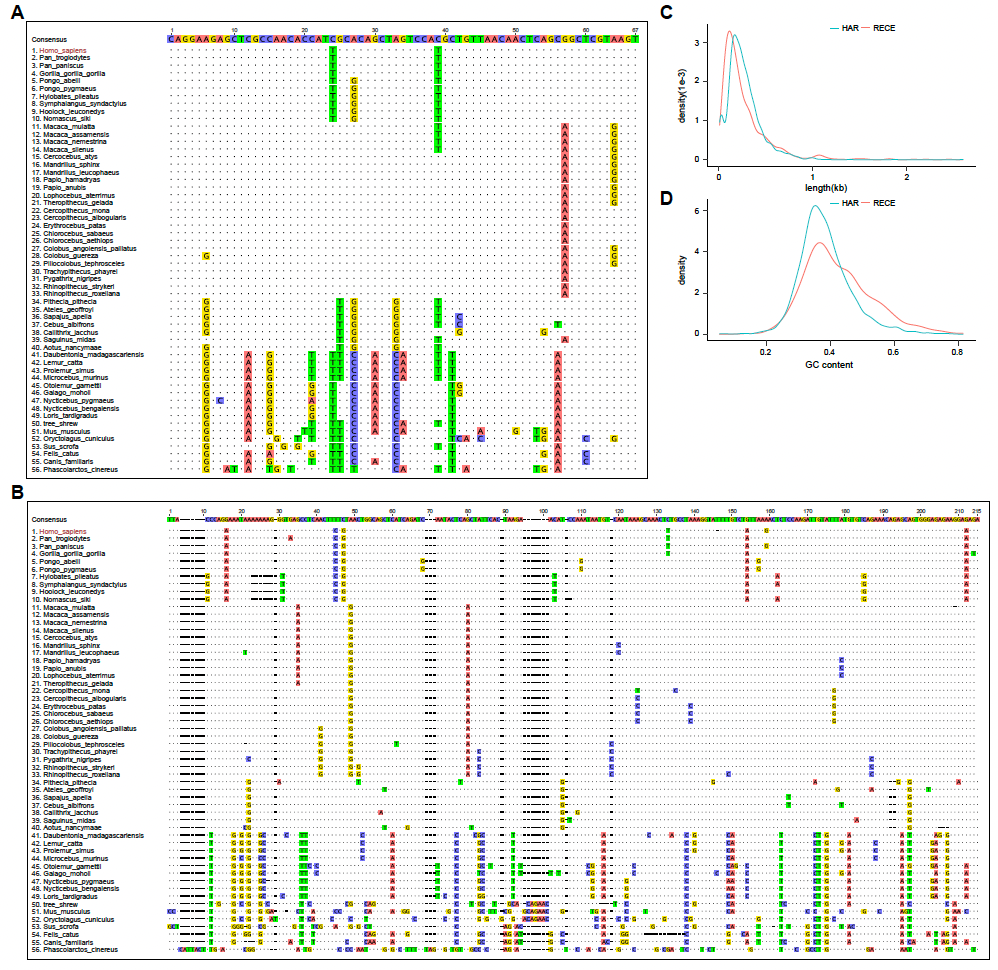


**Figure S1. The length and GC content of RECEs**. **A-B**. Examples of RECEs. **C-D**. Density plots showing the length and GC content of RECEs and HARs.

**Figure S2**.


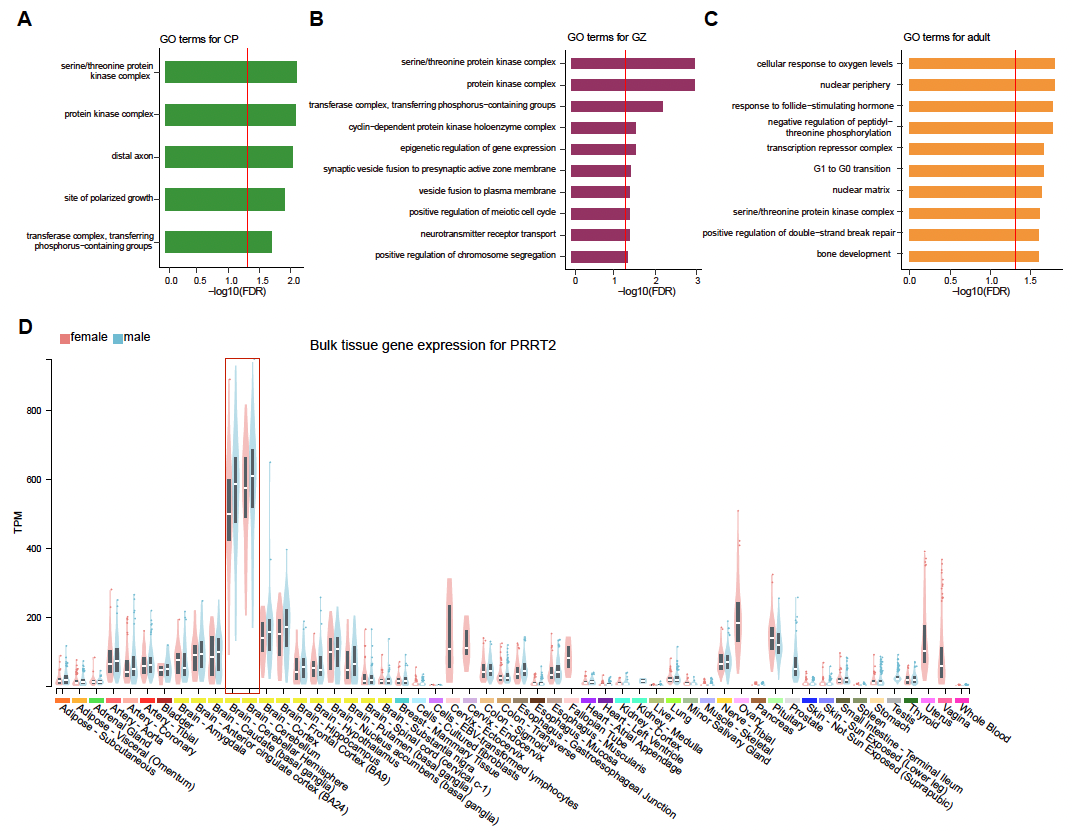


**Figure S2. The length and GC content of RECEs**. **A-C**. Gene ontology (GO) analysis for genes assigned to RECEs in CP (**A**), GZ (B) and adult brains (**C**). The red line denotes FDR = 0.05. **D**. The barplot showing the expression levels of *PRRT2* across different tissues from the GTEx database.

**Figure S3**.


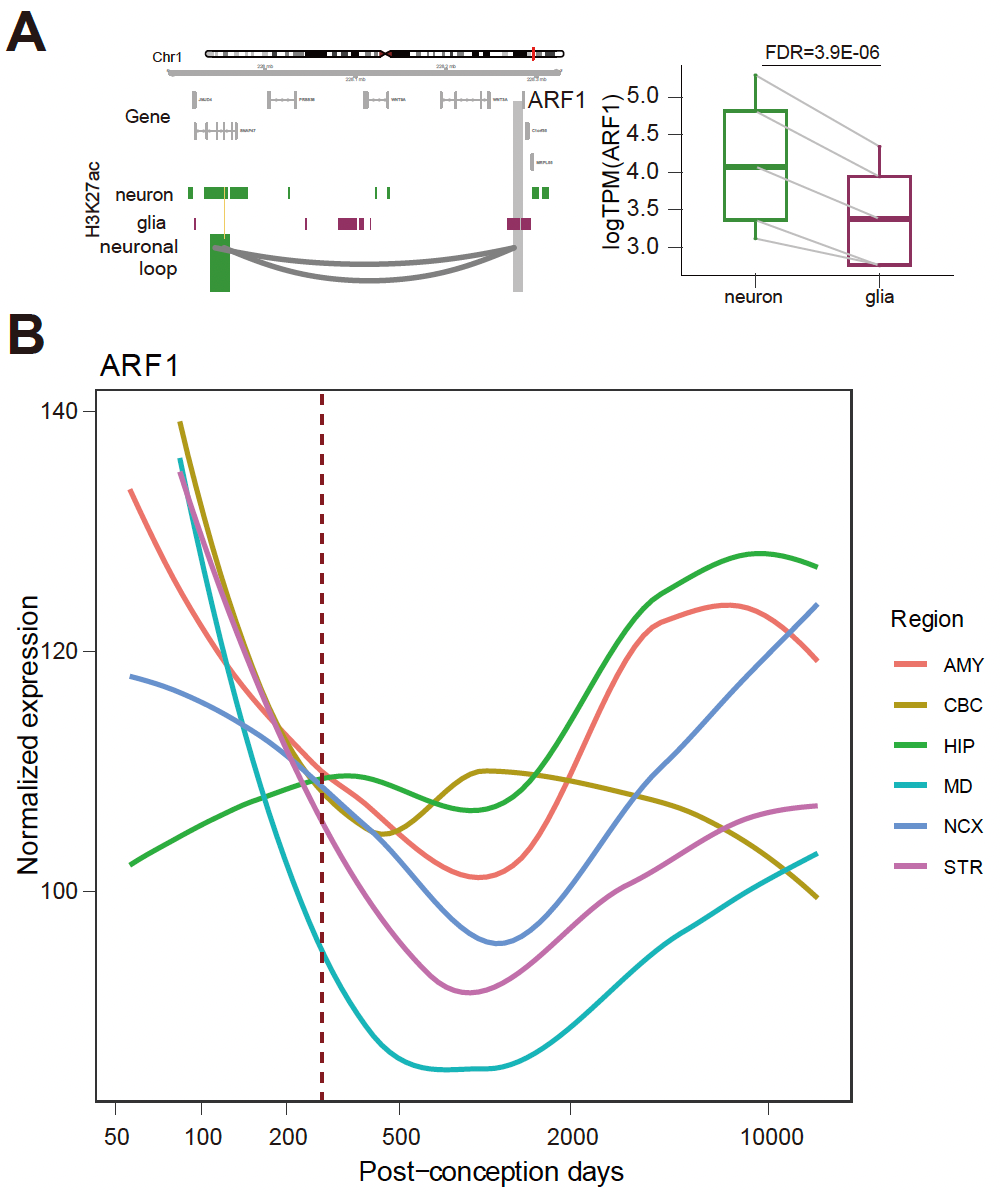


**Figure S3. The expression levels of ARF1 during human brain development**. **A**. *ARF1* is engaged with neuron-specific H3K27ac peaks via enhancer-promoter interactions. The regions that interact with the gene promoter (grey) are highlighted in green (neuron). Red bars represent RECEs. Boxplots on the right show expression levels of *ARF1* in neurons and glia. The FDR was calculated by DESeq2. **B**. Expression trajectories of *ARF1* in human fetal and adult brains during the course of human brain development. The expression level of a sample at a specific developmental time point is represented by the median expression level with correction against the background of all expressed genes in human brain transcriptomes. The expression data were obtained from previous studies^17^.

**Figure S4**.


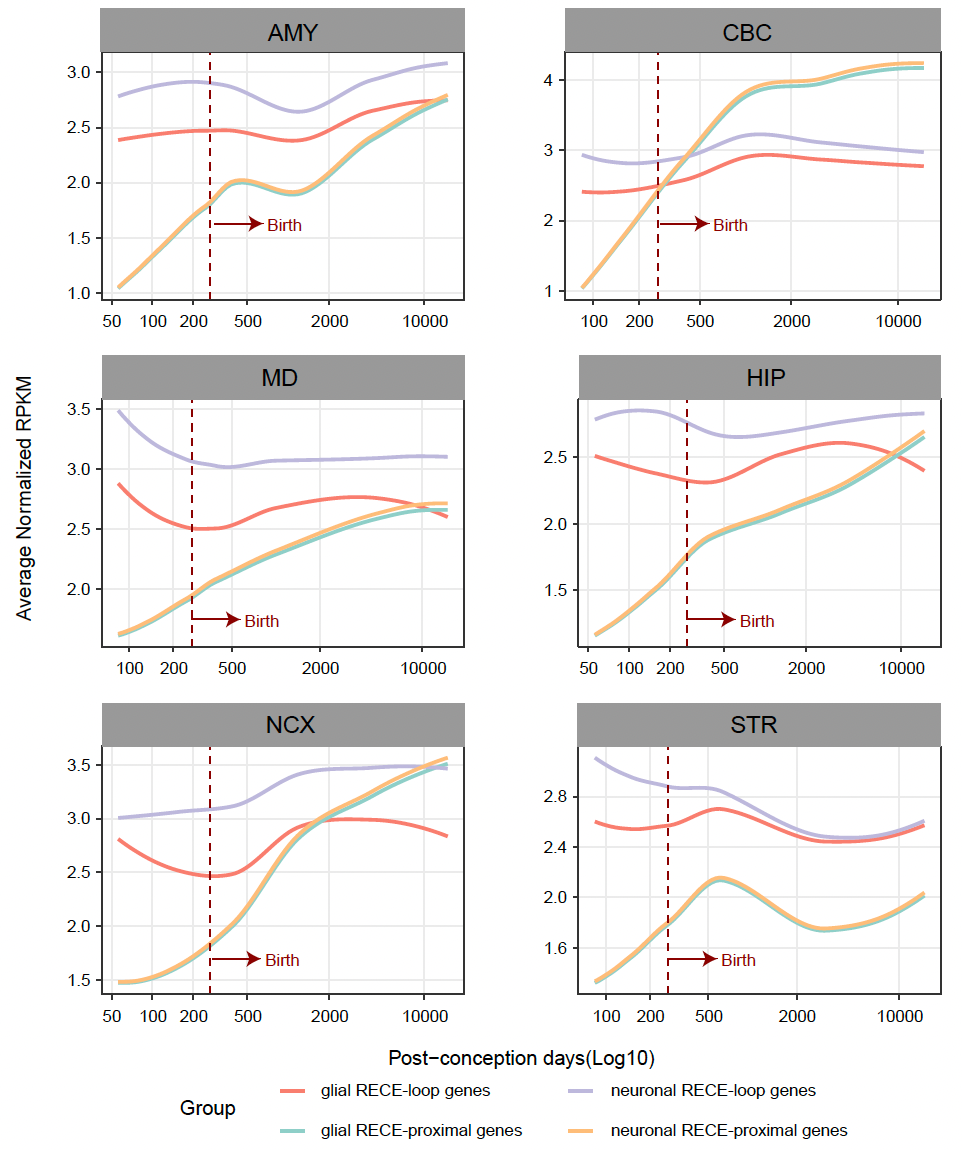


**Figure S4. Expression trajectories of neuron- and glial-expressed genes linked to RECEs in human brain development**. Expression trajectories of neuronal RECE-loop genes, neuronal RECE-proximal genes, glial RECE-loop genes and glial RECE-proximal genes in six brain regions during the course of human brain development. The expression level of genes in samples at specific developmental time points is represented by the median expression level with correction against the background of all expressed genes in human brain transcriptomes. The expression data were obtained from previous studies^18^.

**Figure S5**.


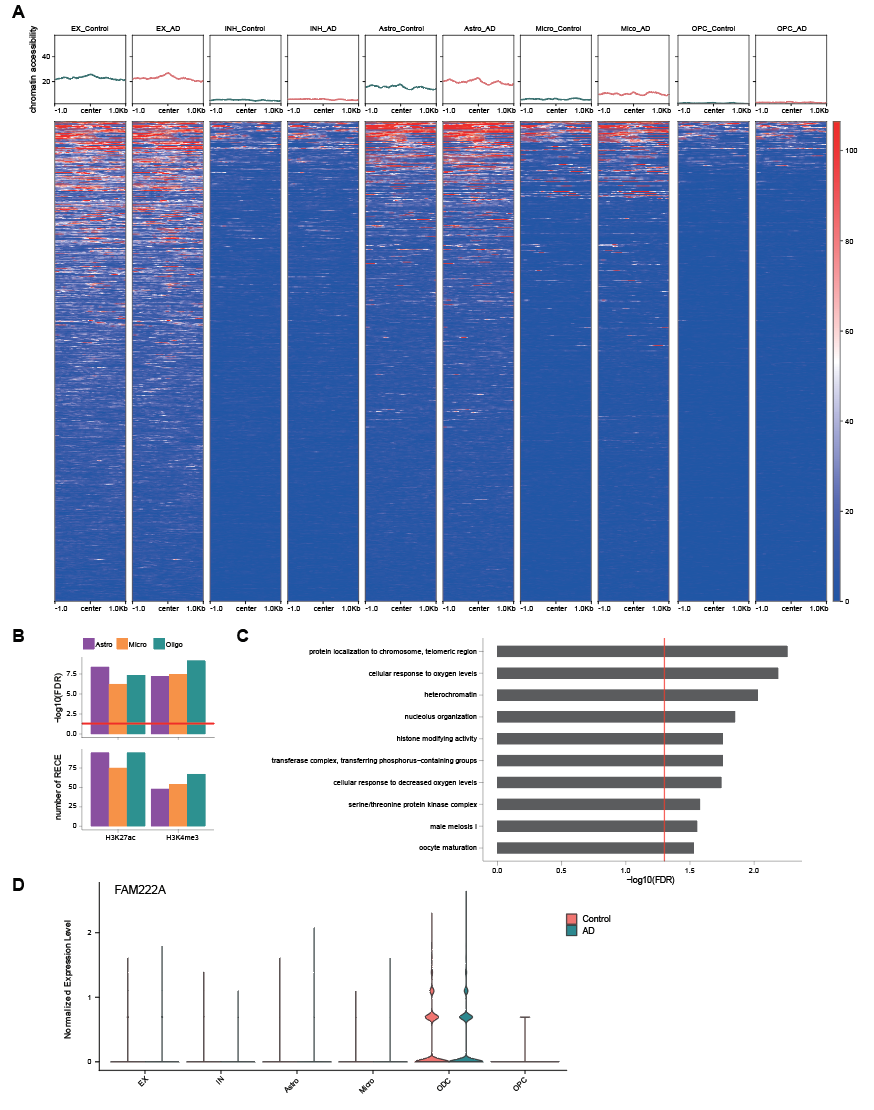


**Figure S5. Characteristics of oligodendrocyte-expressed genes linked to RECEs**. **A**. Chromatin accessibility of RECEs in EX, INH, Astro, Micro and OPC based on snATAC-seq from individuals with and without AD. **B**. Analysis of regulatory activity of RECEs in Astro, Micro and Oligo based on H3K4me3 and H3K27ac ChIP-seq. **C**. GO analysis for genes assigned to RECEs in Oligo. The red line denotes FDR = 0.05. **D**. The violinplot showing the single-cell expression level of FAM222A in EX, INH, Astro, Micro, Oligo and OPC based on snRNA-seq^49^. EX, Excitatory neurons; INH, Inhibitory neurons; Astro, Astrocytes; Micro, Microglia; Oligo, Oligodendrocytes; OPC, Oligodendrocyte progenitor cells.

**Figure S6**.


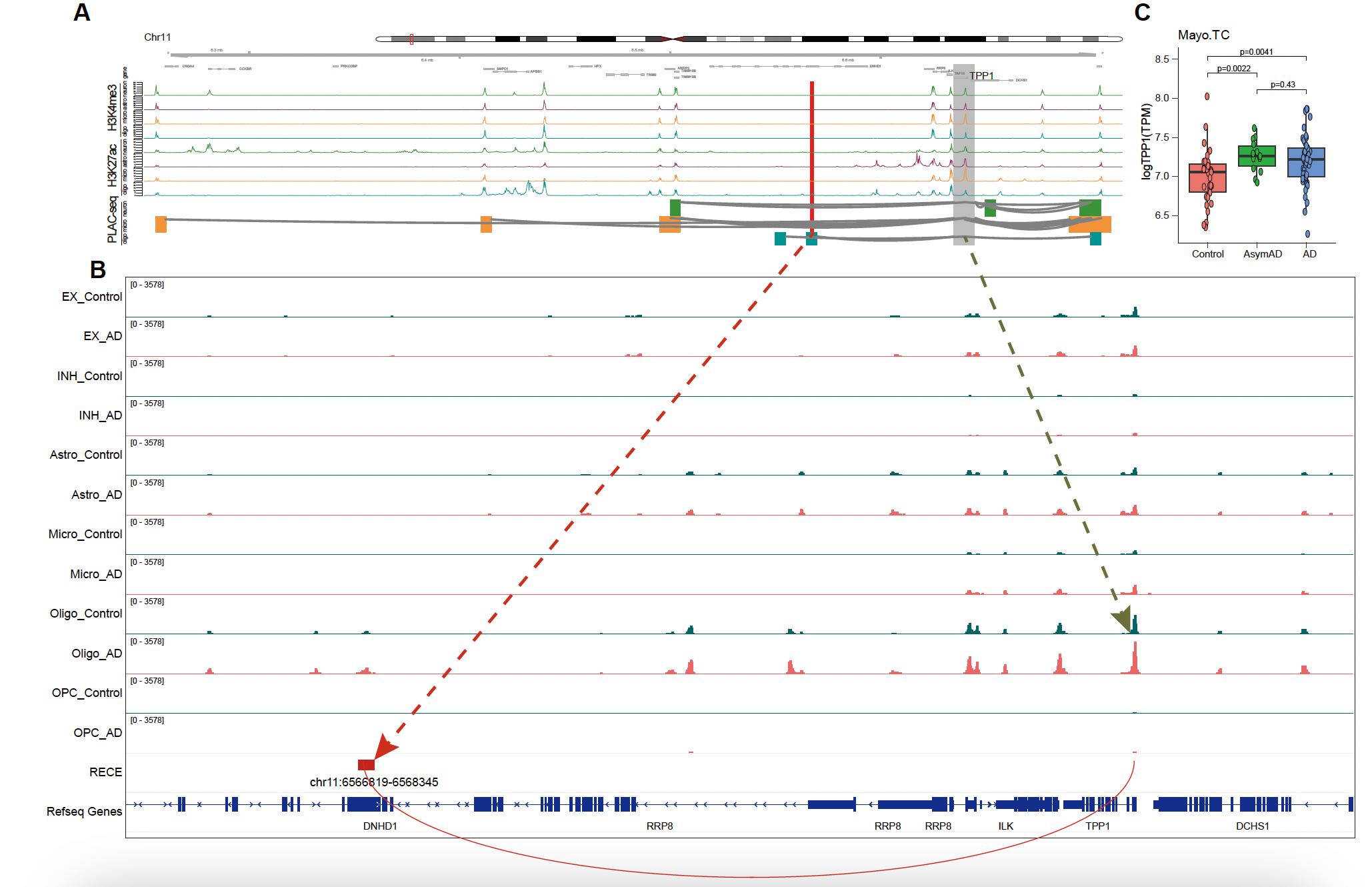


**Figure S6. The *TPP1* gene may be transcriptionally activated by a RECE**. **A**. *TPP1* is engaged with neuron-, Astro- and Micro-specific H3K27ac peaks via enhancer-promoter interactions. The regions that interact with the gene promoter (grey) are highlighted in green (neuron), yellow (Astro), and blue (Micro). Red bars represent RECEs. **B**. The genomic visualization showing the genomic coordinates of one RECE interacting with the *TPP1* promoter. The red rectangle denotes the location of the RECE. **C**. Boxplots in the right show expression levels of *TPP1* in Control, AsymAD and AD brain tissues (<http://swaruplab.bio.uci.edu:3838/bulkRNA/>). EX, Excitatory neurons; INH, Inhibitory neurons; Astro, Astrocytes; Micro, Microglia; Oligo, Oligodendrocytes; OPC, Oligodendrocyte progenitor cells.

**Supplementary Information**

**Table S1.  Coordinates of RECEs in the hg19 and hg38 genome assemblies.**

**Table S2.  Transcription factor binding sites in RECEs.**

**Table S3. Coordinates of RECEs in gene promoters or in distal enhancers interacting with gene promoters in human CP, GZ and adult brain tissues.**

**Table S4. Correlation coefficient and significance between WGCNA modules and development time.**

**Table S5. Coordinates of RECEs in gene promoters or in distal enhancers interacting with gene promoters in neurons and glia.**

**Table S6. Coordinates of RECEs in gene promoters or in distal enhancers interacting with gene promoters in oligodendrocytes**
